# Severe pain as a possible cause of dropped head syndrome that was attenuated after amputation of an ischemic lower limb

**DOI:** 10.1186/s13104-016-1952-3

**Published:** 2016-03-02

**Authors:** Satoshi Maki, Masao Koda, Takeo Furuya, Kazuhisa Takahashi, Masashi Yamazaki

**Affiliations:** Department of Orthopaedic Surgery, Chiba University Graduate School of Medicine, 1-8-1 Inohana, Chuou-ku Chiba, 260-8670 Japan; Department of Orthopaedic Surgery, Faculty of Medicine, University of Tsukuba, 1-1-1 Tennodai, Tsukuba, 305-8575 Japan

**Keywords:** Dropped head syndrome, Isolated neck extensor myopathy, Pain

## Abstract

**Background:**

Dropped head syndrome (DHS) is defined as weakness of the neck extensor muscles causing a correctable chin-on-the-chest deformity. Here we report the case of a patient with severe pain from lower leg ischemia showing DHS whose symptoms were attenuated by pain relief after amputation of the severely ischemic lower leg. To our knowledge this is the first report indicating that severe pain can cause DHS.

**Case presentation:**

A 64-year-old Asian woman was referred to our department with a 1-month history of DHS. She also suffered from severe right foot pain because of limb ischemia. She began to complain of DHS as her gangrenous foot pain worsened. She had neck pain and difficulty with forward gaze. We found no clinical or laboratory findings of neuromuscular disorder or isolated neck extensor myopathy. We amputated her leg below the knee because of progressive foot gangrene. Her severe foot pain resolved after the surgery and her DHS was attenuated.

**Conclusion:**

Severe pain can cause DHS. If a patient with DHS has severe pain in another part of the body, we recommend considering aggressive pain relief as a treatment option.

## Background

Dropped head syndrome (DHS) is defined as apparent weakness of the neck extensor muscles that causes difficulty in lifting the head against gravity, which results in a correctable chin-on-the-chest deformity. DHS can impair activities of daily living. The main symptoms of DHS include impairment of forward gaze, neck pain, and myelopathy or radiculopathy, or both. DHS is most commonly associated with neuromuscular disorders such as myasthenia gravis, polymyositis, and amyotrophic lateral sclerosis [1–3]. Whereas, in some patients with a dropped head, no specific abnormalities are found and they have what is called isolated neck extensor myopathy [4]. Here we report the case of a patient with lower leg ischemia and severe pain presenting with DHS, and whose symptoms were attenuated by pain relief after amputation of the severely ischemic lower leg. To our knowledge, this is the first report of severe pain causing DHS.

## Case presentation

**Fig. 1:**
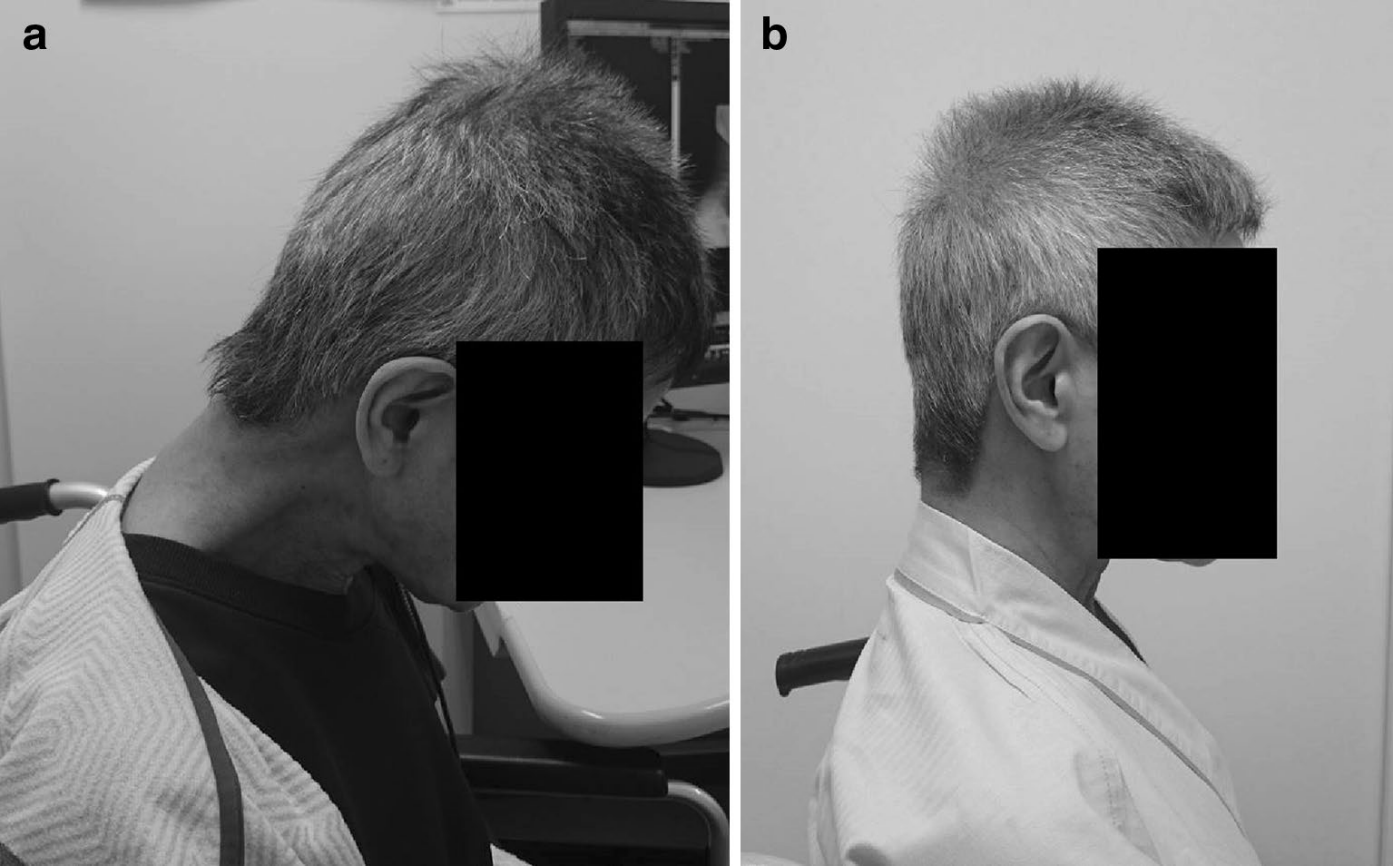
Comparison of the patient’s posture at diagnosis and 3 weeks after surgery. **a** Photograph of the patient at her first visit to our department, which shows apparent severe weakness of the neck extensor muscles. **b** Photograph of the patient after surgery, which shows improvement of neck posture

**Fig. 2:**
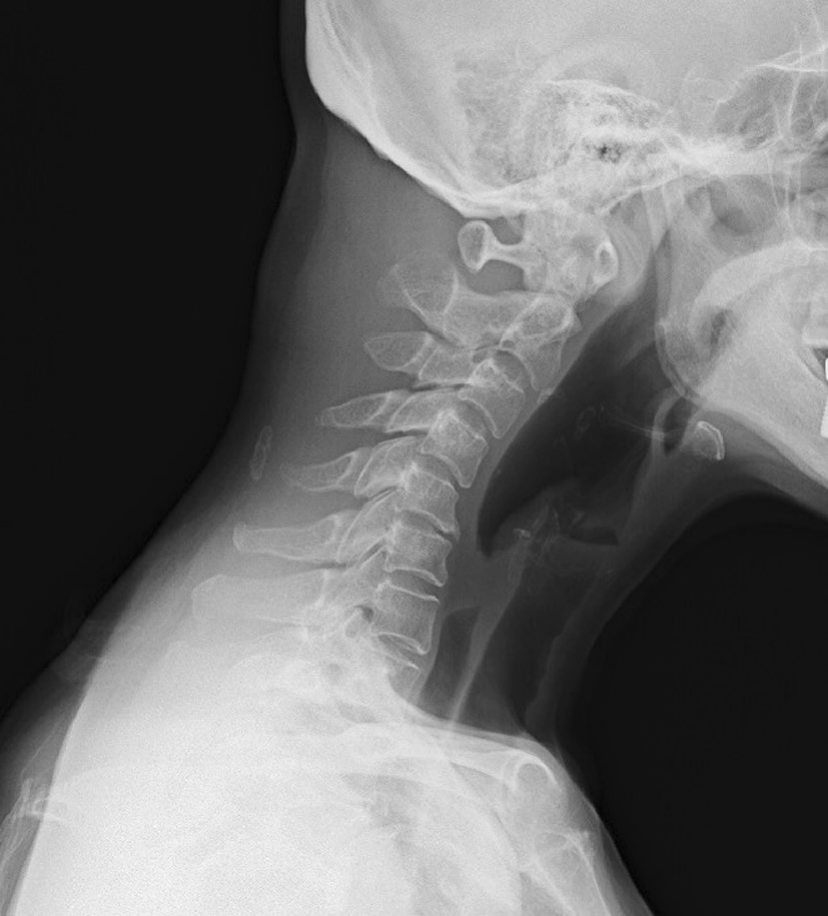
Lateral radiograph of cervical spine in a neutral position. Plain radiography shows severe cervical kyphosis at the patient’s first visit to our department

A 64-year-old Asian woman was referred to our department with a 1-month history of DHS. She also suffered from severe right foot pain because of limb ischemia related to systemic sclerosis. She had a history of systemic sclerosis that was diagnosed 30 years earlier and was classified as having limited cutaneous systemic sclerosis. Cutaneous fibrosis was limited to her hands without involvement of her head or neck. There was no family history of any neuromuscular disease. One month before visiting our hospital she developed right foot gangrene. Although she had Raynaud’s phenomenon and ulcerations in her fingers, she had had no symptom in her foot before she had gangrene. Pain in her right foot was so severe that she used tramadol and NSAIDs as painkillers. She began to complain of DHS as her foot pain worsened. She had difficulty in keeping her head straight and her gaze forward. Clinical examination revealed severe weakness of her neck extensor muscles. Her neck deformity was flexible and easily corrected passively (Fig. 1a). There were no neurological abnormalities including sensory disturbance or muscle weakness in her extremities. Plain radiographs of her cervical spine revealed only mild degenerative changes and severe cervical kyphosis (Fig. 2). Cervical magnetic resonance imaging showed no spinal canal stenosis and no abnormal signal change in the cervical paraspinal muscles. Computed tomographic angiography showed occlusion of bilateral anterior tibial arteries and peroneal arteries, and development of collaterals. Despite intensive conservative therapy including alprostadil, argatroban, and sarpogrelate, lower extremity ischemia progressed. Therefore, we decided to amputate her leg below the knee under general anesthesia. Her postoperative course was uneventful. Her severe foot pain was markedly resolved after the surgery. At the postoperative follow-up visit 3 weeks after surgery, her DHS was significantly attenuated (Fig. 1b). Her forward vision and neck pain were markedly improved.

The feature of the present patient was attenuation of her DHS after pain relief by amputation of her severely ischemic limb. The clinical course of the present case suggests that severe pain from her ischemic limb caused the DHS.

The patient had a medical history of systemic sclerosis which is also known as scleroderma. There is one case report of a patient with systemic sclerosis who developed DHS, but showed no improvement in a 2-year follow up [5]. Considering the DHS in our patient resolved within 2 months from onset regardless of the state of the systemic sclerosis, her DHS was unrelated to either systemic sclerosis or other neuromuscular disease. The vast majority of cases of isolated neck extensor myopathy never resolve spontaneously, so it is less likely that she had isolated neck extensor myopathy [6]. We also found no clinical or laboratory evidence of myositis [7]. The onset of her DHS coincided with worsening of foot pain and improvement of DHS coincided with pain relief. The cause and the pathogenesis of the DHS remain unclear, but there is a possibility that severe pain caused the DHS. The indirect lines of evidence suggest that severe pain can be a cause of DHS. Although it may be rare, if a patient with DHS has severe pain, we recommend considering the possibility that severe pain is a cause of the DHS after excluding neuromuscular disease. The underlying mechanism by which pain causes DHS is unclear and warrants investigation.

## Conclusion

Severe pain can cause DHS. If a patient with DHS has severe pain in another part of the body, aggressive pain relief should be considered as a treatment option.

## Consent

Written informed consent was obtained from the patient for publication of this case report and any accompanying images.

## Abbreviation

DHS: dropped head syndrome

## References

[1] Suarez GA, Kelly JJ (1992). The dropped head syndrome. Neurology.

[2] Umapathi T, Chaudhry V, Cornblath D, Drachman D, Griffin J, Kuncl R (2002). Head drop and camptocormia. J Neurol Neurosurg Psych.

[3] Finsterer J (2004). Dropped head syndrome in mitochondriopathy. Eur Spine J.

[4] Katz JS, Wolfe GI, Burns DK, Bryan WW, Fleckenstein JL, Barohn RJ (1996). Isolated neck extensor myopathy: a common cause of dropped head syndrome. Neurology.

[5] Rosato E, Rossi C, Salsano F (2009). Dropped head syndrome and systemic sclerosis. Joint Bone Spine.

[6] Sharan AD, Kaye D, Charles Malveaux WMS, Riew KD (2012). Dropped head syndrome: etiology and management. J Am Acad Orthop Surg.

[7] Gaeta M, Mazziotti S, Toscano A, Rodolico C, Mazzeo A, Blandino A (2006). “Dropped-head” syndrome due to isolated myositis of neck extensor muscles: MRI findings. Skeletal Radiol.

